# Interprofessional communication - a concept analysis inspired by Schwartz-Barcott and Kim´s hybrid model

**DOI:** 10.1186/s12913-026-14180-3

**Published:** 2026-02-17

**Authors:** Ingela Rudberg, Annakarin Olsson, Charlotta Thunborg, Martin Salzmann-Erikson

**Affiliations:** 1 Department of Health and Caring Sciences, Faculty of Health and Occupational Studies, University of Gävle, Gävle, SE-801 76 Sweden; 2 Division of Clinical Geriatrics, Department of Neurobiology, Care Sciences and Society, Karolinska Institutet, Stockholm, Sweden

**Keywords:** Interprofessional communication, Interprofessional collaboration, Concept analysis, Hybrid model, Literature review

## Abstract

**Aim:**

This concept analysis aims to describe and define interprofessional communication within healthcare contexts by synthesising empirical evidence and existing literature.

**Design:**

Utilising Schwartz-Barcott and Kim´s hybrid model, the study integrates theoretical exploration with empirical data analysis.

**Methods:**

A comprehensive literature review and dictionary references inform the selection of theoretical frameworks. Data collection involves screening articles from databases including CINAHL, PSYCHINFO, WEB OF SCIENCE, SCOPUS, and PUBMED, resulting in 37 articles for review. Secondary analysis includes observational studies and focus groups conducted in psychiatric outpatient units, integrating theoretical and empirical insights for a nuanced understanding of communication complexities in healthcare settings.

**Results:**

Theoretical exploration reveals the multifaceted nature of interprofessional communication, emphasising information sharing, collaboration, and decision-making. Antecedents, including education, logistical challenges, legal frameworks, and cultural dynamics, are identified. Empirical findings underline the importance of competence, curiosity, trust, cooperation, and clarity in communication. Influential factors such as leadership, cultural understanding, informal communication, transparency, and hierarchy emerge.

**Conclusions:**

The synthesis highlights the central role of communication in healthcare and emphasises its impact on outcomes. Addressing communication barriers and promoting facilitators is important to supportive work environments and patient safety. Further research is necessary to deepen understanding and improve communication effectiveness in health care.

**Supplementary Information:**

The online version contains supplementary material available at 10.1186/s12913-026-14180-3.

## Background

In healthcare, interprofessional communication (IPC) is widely described as central to patient outcomes and staff well-being. At the same time, terminology in this field is often overlapping, with concepts such as interpersonal communication, interprofessional collaboration, and interprofessional communication used inconsistently across the literature [1, 2]. The term *“Interprofessional”* refers to collaborative work across professional boundaries aimed at promoting patient care and well-being, preventing and treating disease, and reducing the negative consequences associated with communication errors [3–5].

Although interprofessional communication and interprofessional collaboration are closely related and often used interchangeably, they represent analytically distinct concepts. Interprofessional communication refers to the communicative processes, specifically the ongoing exchange of information through verbal, nonverbal, and digital channels across professional boundaries [6–8]. In contrast, interprofessional collaboration denotes a broader practice configuration and relational outcome, encompassing shared goals, role clarity, and coordinated decision-making over time [3, 5]. From this perspective, IPC can be understood as the operational mechanism through which collaboration is enacted and sustained, rather than as a collaborative outcome in itself. Clarifying this distinction is important for conceptual precision and for distinguishing communication-specific attributes from those associated with collaboration, teamwork, or coordination.

Interprofessional communication is inherently multifaceted, involving communicative processes embedded in collaborative work and information exchange through verbal and nonverbal channels [6, 7, 9]. These processes influence professional relationships, illustrating the dynamic nature of human interaction [6, 7]. Moreover, interprofessional communication extends beyond traditional face-to-face interactions to encompass electronic and digital media, reflecting the increasing role of technology in healthcare communication practices [8].

The present concept analysis focuses specifically on interprofessional communication within healthcare contexts. While the term *“interprofessional”* is applicable across various contexts, including education, public communication, program implementation, and legal settings [10–13], this study is deliberately delimited to healthcare to support concept development grounded in clinical practice.

Previous reviews have examined interprofessional communication in healthcare and highlighted its relevance for information exchange and professional relationships [3, 14, 15]. Across both reviews and primary studies, research on interprofessional communication has predominantly focused on acute care and surgical settings and has often been limited to interactions between physicians and nurses [16–22]. As a result, less is known about how interprofessional communication functions across a wider range of professional groups and in other clinical contexts, such as psychiatric outpatient care.

From a concept development perspective, psychiatric outpatient care constitutes a particularly informative empirical context because IPC in this setting often precedes, exceeds, or unfolds independently of formalized interprofessional collaboration. Communication is typically continuous, relationship-based, and distributed across professional, organizational, and service boundaries over time rather than within discrete clinical encounters. This makes psychiatric outpatient care well-suited for examining IPC as a process in its own right and for empirical refining conceptual boundaries between communication and related constructs such as collaboration or coordination.

To address this need, the present study employs Schwartz-Barcott and Kim´s hybrid model for concept analysis [23]. The hybrid model integrates a theoretical phase, involving systematic exploration of existing literature, with a fieldwork phase based on empirical observation, followed by an analytical phase in which theoretical and empirical insights are iteratively integrated.

Unlike concept analysis methods that rely primarily on theoretical synthesis, such as Walker and Avant´s technique [24] or Rodgers´ evolutionary approach [25], the hybrid model allows for continuous movement between theory and empirical data, supporting context-sensitive understanding of complex concepts [23]. In addition, the hybrid model incorporates a phenomenological sensitivity that facilitates engagement with participants’ lived experiences, aligning with broader phenomenological perspectives on understanding human experience in context [26]. This approach enhances the validity and relevance of concept development within nursing and healthcare research.

By examining interprofessional communication in psychiatric outpatient care and situating these findings within the broader body of healthcare communication literature, the present study seeks to broaden the scope of existing research. Beyond conceptual clarification, the analysis is intended to inform clinical practice and to provide a foundation for future research on interprofessional communication and its implications for patient and clinician outcomes.

Aim: This concept analysis aims to describe and define interprofessional communication within healthcare contexts by synthesising empirical evidence and existing literature.

## Method

### Study design - the hybrid model

**Fig. 1:**
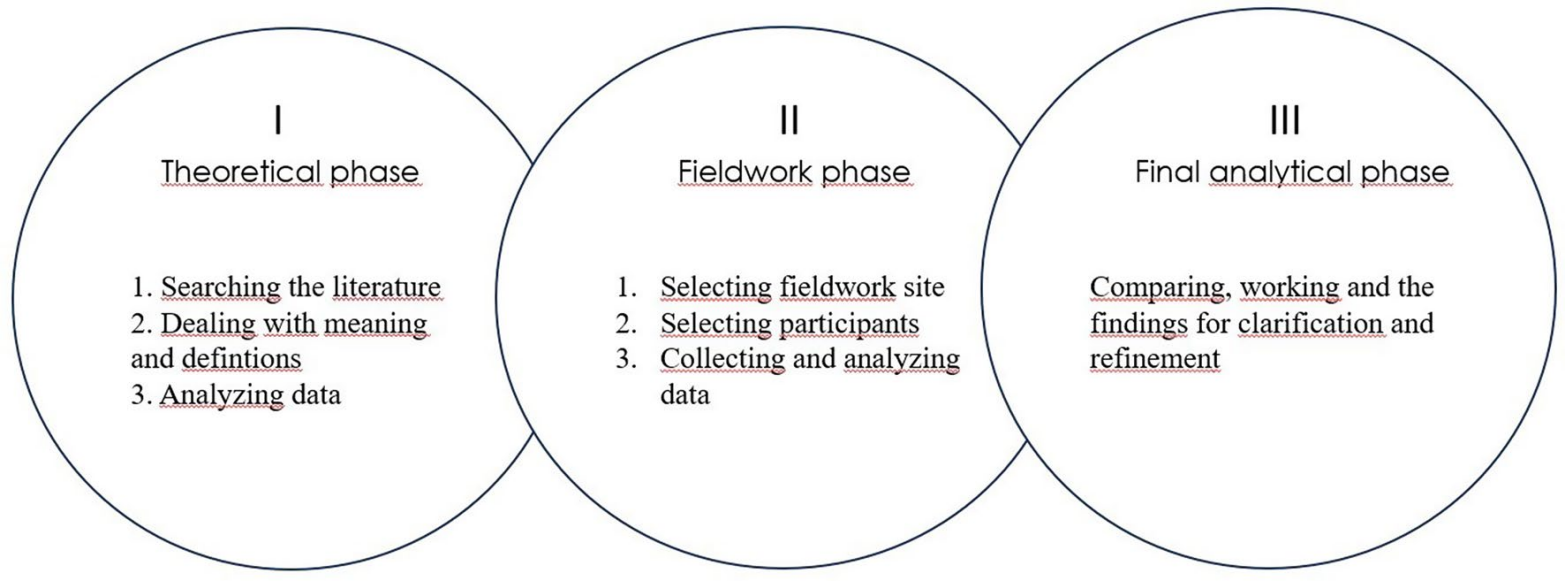
Schematic overview of the study design using Schwartz-Barcott and Kim´s hybrid model

Schwartz-Barcott and Kim´s [23] hybrid model comprises three sequential phases: theoretical exploration, fieldwork, and analytical phases, each marked by unique objectives and methodological approaches, as illustrated in Fig. 1. To enhance transparency, the Prisma-ScR checklist [27] was applied. As scoping and literature reviews are not eligible for PROSPERO registration, no protocol registration was completed.

### Data collection and procedure

To develop a comprehensive understanding of interprofessional communication, an extensive literature search was conducted on November 29, 2023, across five databases (CINAHL, PsycINFO, Web of Science, SCOPUS, and PubMed), complemented by dictionary searches to support conceptual clarity. Tailored search strategies and language restrictions were applied. After duplicate removal, 1,410 unique articles were screened using Rayyan, a web-based software platform for systematic reviews.

Inclusion criteria required explicit use of the term *interprofessional communication* in the title and a focus on healthcare professionals within care-providing settings. Only full-text sources published from 2010 onward were included. This strict terminology requirement may have limited conceptual breadth and introduced potential terminology bias. Studies outside healthcare, educational settings, and ad hoc publications (e.g., conference proceedings, workshops, book chapters, grey literature) were excluded.

To ensure the reliability of our study, we employed a meticulous screening process involving four researchers. Each researcher reviewed half of the total 1410 articles, resulting in dual independent assessments for each article. Disagreements were resolved through discussion and revisiting the eligibility criteria. After full-text review, 37 articles were included as summarised in Fig. 2 based on the PRISMA flowchart. Engaging two independent groups in collaborative investigations and transparently resolving conflicts ensured the trustworthiness of our study.

### PRISMA flowchart

**Fig. 2:**
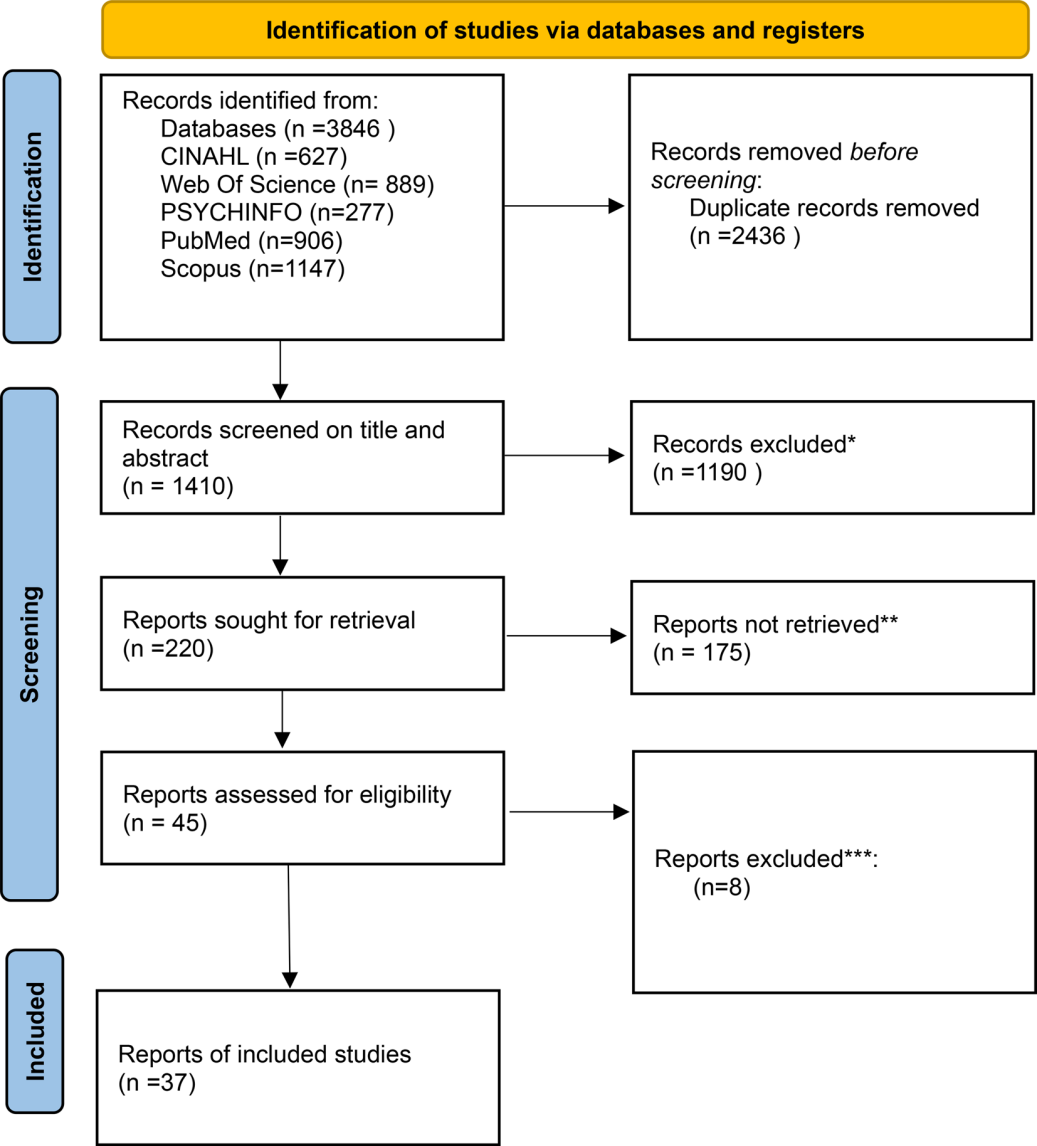
The flowchart detailing the decision-making process for the selection, critical appraisal, and data extraction during the theoretical phase. * Exclusions were based on our criteria, requiring “interprofessional communication” to be present in both the title and abstract or publication before 2010 ** were unrelated to health care, book chapters, educational settings, and discussions outside of care settings. *** Conference reports and full texts that did not focus on INTERPROFESSIONAL COMMUNICATION

### Fieldwork phase

Following the theoretical phase, the study proceeded to the fieldwork phase, drawing on empirical raw-data from previous studies. These datasets were re-examined at the raw-data level (transcripts, field notes), allowing an independent conceptual analysis distinct from the original studies´ aims and interpretations. Specifically, we utilised data from a secondary analysis of observational studies [28] and focus groups [29] conducted within psychiatric outpatient units. The primary studies focused broadly on interprofessional communication in psychiatric outpatient care, whereas the current study applies the hybrid model to conceptually analyse the phenomenon, thus extending the empirical material beyond its initial analytic purpose.

The dataset comprised over 100 h of observations from team meetings, treatment conferences, and assessment sessions at a medium-sized psychiatric outpatient unit in central Sweden. In addition, five semi-structured focus groups with clinicians from multiple professional backgrounds were conducted. The outpatient units were selected based on practicality and geography, and none of the authors had prior relationships with the setting or clinicians. All data were audio-recorded, de-identified, and securely stored. Further procedural details are reported in Rudberg et al. [28, 29].

### Data analysis

The final analytical phase involved comparing and integrating insights from the theoretical and fieldwork phases. The analysis began with a thorough literature review and text analysis. In the theoretical phase, literature was read repeatedly, and meaning units were categorised into antecedents, attributes, and consequences, drawing on Walker and Avant´s [24] structure.

In the empirical phase, the secondary analysis focused on attributes and antecedents, as consequences were less prominent in the available data. Meaning units were identified and coded by the first author, with interpretations and categorizations discussed with co-authors to enhance reflexivity and mitigate bias. Although the first author conducted the original data collection and primary analyses, the current study constitutes a secondary interpretive analysis: all datasets were re-examined at the level of raw data, and the analytic focus was explicitly conceptual rather than reproducing prior findings. This approach enabled a new, independent interpretation of IPC within psychiatric outpatient care, while discussions with co-authors and iterative reflection against the theoretical framework helped mitigate the risk of confirmation bias or analytic carryover. Results from both phases were then systematically compared to refine and strengthen the conceptual understanding. Discrepancies between theoretical and empirical findings were resolved through discussion and by referring to the conceptual framework, ensuring consistent integration across phases. Following Madden´s [30] approach, this iterative and interpretive process allowed the development of a definition consistent with both existing literature and participants´ lived experiences.

### Rigor

To ensure analytic independence, all empirical datasets were re-examined at the level of raw data rather than relying on prior interpretations. This enabled a fresh examination of the material and a conceptual interpretation aligned with the aims of the hybrid model. The iterative comparison between theoretical and empirical phases, guided by the hybrid model, supported systematic integration and refinement of conceptual insights. Rigor was further strengthened by analytic transparency, including reflexivity, which was maintained through documentation of analytical decisions and critical discussion of interpretations and critical discussion within the research team. Details of the literature search strategy, including databases and search strings, are provided in Appendix 1. In addition, rigor was further supported through systematic mapping across the included articles, enabling transparent comparison of conceptual usage to individual sources (see Table 1).

**Table 1:**
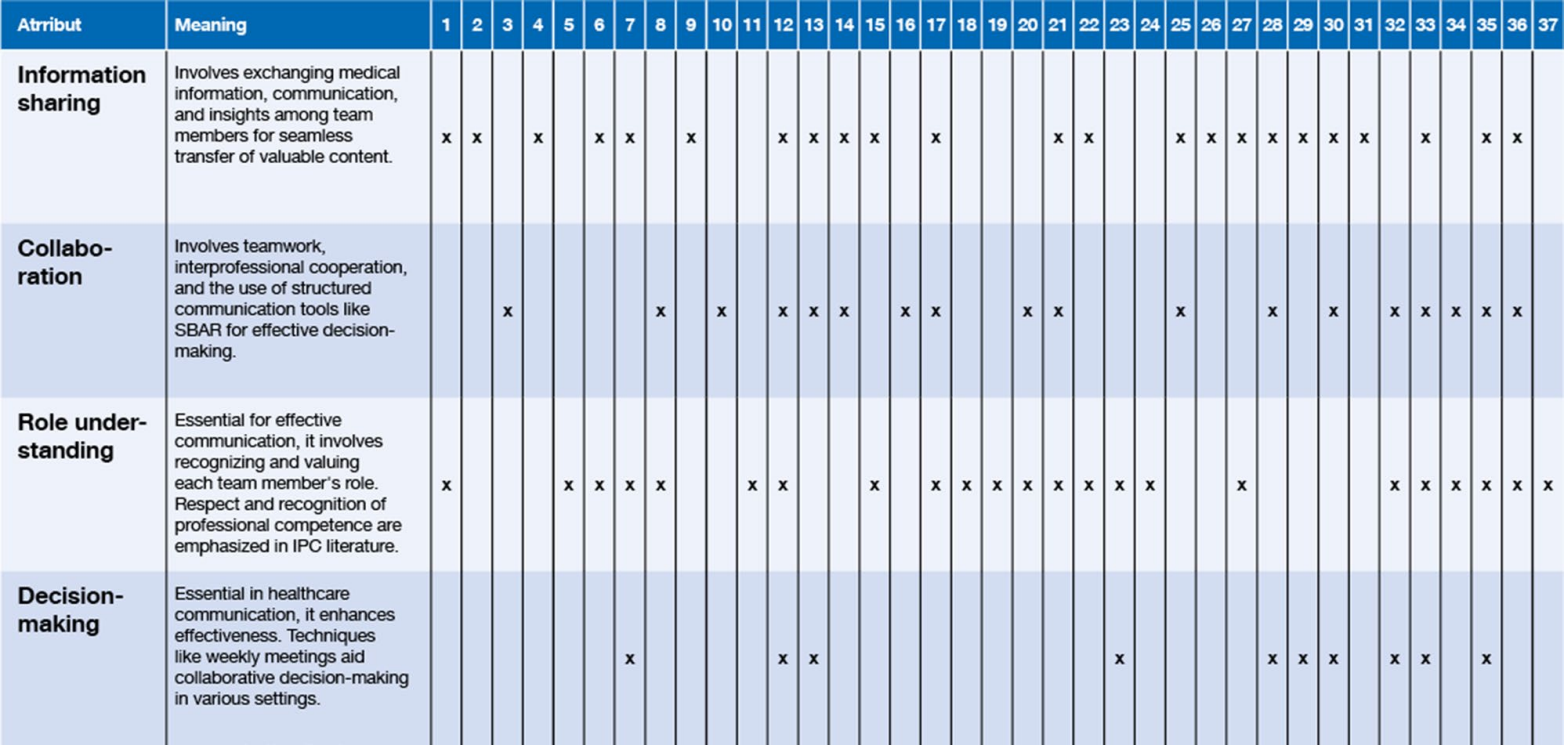
Attributes summary table, article no. 1–37, see Appendix 2

### Ethical considerations

This study was conducted in accordance with the ethical principles of the Declaration of Helsinki [31]. A secondary analysis design was employed, involving the reuse of pre-existing qualitative data from previous studies (semi-structured interviews and field notes), thereby avoiding additional data collection and minimizing potential risks to participants [32]. By adopting this methodological approach, we not only uphold ethical standards but also maximize the utility of existing resources.

The secondary analysis was conducted using data for which informed consent had been obtained in the original studies. The original data collection was approved by the Swedish Ethical Review Authority (No. 2022-00023-001), and all data were collected in accordance with that approval. No additional consent was sought, as the analysis did not involve new data collection or re-contact with participants. An application for ethical review of the secondary analysis was submitted to the Swedish Ethical Review Authority. The Authority determined that the project did not fall within the scope of the Swedish Ethics Review Act and therefore did not require formal ethical approval. However, the Authority issued an advisory opinion (No. 2024-02699-01) stating that it had no ethical objections to the study.

To ensure confidentiality and data protection, all data were handled in accordance with applicable data protection regulations. The material was de-identified before analysis; participants were referred to only by professional role, and no direct personal identifiers were included in the secondary analysis.

The first author´s background as a psychiatric nurse is acknowledged as a valuable contextual insight, contributing to reflexivity and ethical awareness throughout the research process.

## Results

The results are organised into antecedents, attributes, and consequences of interprofessional communication. Based on their primary analytical function rather than their potential occurrence across different phases of communication processes. Antecedents refer to contextual or structural conditions that must be present prior to IPC and that enable or constrain its emergence. Attributes represent the core characteristics that constitute interprofessional communication itself and describe how the concept is manifested in practice. Consequences denote outcomes or effects that result from the presence or absence of effective interprofessional communication.

Some elements (e.g., leadership, trust, competence, hierarchy) recur across multiple analytical levels in literature and empirical material. These were classified according to their dominant analytical function within this conceptual analysis.

### Theoretical phase characteristics and definition of interprofessional communication

The term *“interprofessional communication”* may not be found in traditional dictionaries like the Cambridge Dictionary, which lacks a direct entry for it [33]. However, individual components of the terms— *“interprofessional”* and *“communication”—*are recognised in various dictionaries. A search for *“interprofessional”* on onelook.com yielded 2 dictionaries, while *“communication”* resulted in 59 dictionaries. Related concepts like *“collaboration”* underscore the interconnectedness of communication and collaboration in professional settings [34–36]. Interprofessional communication emerges as a multifaceted and complex concept encompassing both structural and relational dimensions. Table 1 provides an overview of the attributes identified in the theoretical literature and their occurrence across the included articles. Each attribute is elaborated below.

### Attributes

In this theoretical section, information sharing is a central attribute of interprofessional communication [37–55], encompassing the exchange of medical information [50, 56, 57], communication, and insights among diverse team members. It involves gathering and exchanging valuable information [39, 58, 59] with a seamless transfer of content. This multifaceted exchange extends to consulting, seeking opinions, and nurturing a collaborative environment where healthcare professionals inform each other, establishing a cohesive and well-informed interprofessional communication network [37, 50, 53, 60].

Another pivotal aspect emphasized in interprofessional communication is collaboration, which involves teamwork, interprofessional cooperation [42, 47, 55, 58, 61–63], and application of tools such as structured communication, including the widely recognized SBAR (Situation, Background, Assessment, Recommendation) framework, and decision-making techniques [43, 44, 46, 49, 51–54, 62, 64–67]. The category of communication tools and technologies comprehensively encompasses various tools facilitating interactions, diverse communication channels, and forms. While face-to-face communication is generally favoured, the integration of digital communication tools can be a component of effective interprofessional communication [38, 41, 53]. This involves utilising tools and techniques to facilitate interactions and discussions, covering various communication forms and emphasising both reactivity and outcomes.

Role understanding, central to well-functioning interprofessional communication, concerns acknowledging and appreciating each other´s roles within the care team. Similarly, respect and recognition, involving the valuation of each other´s roles, mutual respect, and acknowledgment of professional competence, consistently emerge throughout the literature on interprofessional communication [37, 39, 40, 42, 45–48, 50, 54, 55, 58, 60, 62, 63, 65, 67–73].

The analysis of interprofessional communication underscores the pivotal role of decision-making [40, 42, 43, 51–54, 58, 63, 70]. This attribute occupies a central position in healthcare communication. Shared decision-making plays an important role in enhancing communication effectiveness among healthcare professionals. Diverse techniques, supported by weekly meetings and structured interactions, are employed to facilitate collaborative decision-making across contexts, including scheduled team rounds, corridor meetings, and collaborative scenarios.

### Antecedents

To understand the broader context of interprofessional communication, it is imperative to consider both antecedent and potential barriers. These barriers encompass a wide range of challenges, including *communication gaps*,* technical barriers*,* time constraints*,* hierarchical pressures*,* and a lack of knowledge sharing* [37, 38, 40, 42–44, 46–48, 51, 52, 55, 58–63, 65–68, 70–72]. The antecedents of interprofessional communication include education and knowledge, logistical challenges, legal and confidentiality factors, and cultural and organizational considerations.

Lack of these pose substantial barriers, considerably impeding the seamless flow of communication. Insufficient education and knowledge may result in communication problems and difficulties in recognising the roles of others [43, 45, 54, 62, 68], leading to the undervaluing of opinions. Logistical challenges, including issues in communication logistics and physical separation [38, 42, 45, 46, 61, 70], contribute to unclear communication and inefficient utilisation of communication channels [38, 53, 58]. Legal factors, especially privacy laws, present challenges in information sharing, contributing to a lack of transparency [37]. Cultural and organizational factors [40, 46, 69], encompassing unprofessional culture and hierarchical pressures, create barriers to role understanding, foster a lack of commitment, and contribute to ineffective communication channels [40, 43, 44, 46–48, 52, 58, 60, 61, 63, 67, 70–72].

Antecedents can serve as facilitators that promote interprofessional communication. Abundant interprofessional communication training, streamlined logistical processes, supportive legal frameworks, and a strong organizational culture create an environment where communication flows smoothly, roles are well-understood, and collaboration is enhanced among healthcare professionals from diverse backgrounds [40, 43, 46–48, 50, 53, 57, 60, 65, 69, 73]. Interprofessional communication is key to fostering a positive and collaborative healthcare system. Antecedents play a key role in sparking this collaboration and bringing practitioners together.

### Consequences

The consequences of interprofessional communication, shed light on its influence on healthcare outcomes involving enhanced patient safety, effective care interventions, and improved team collaboration [49, 50, 58, 71, 72]. Conversely, challenges may arise, such as communication issues, treatment delays, and information discrepancies [47, 62]. Understanding the consequences it provides a foundation for further exploration and practical application in healthcare settings.

While the theoretical phase establishes the conceptual foundations of interprofessional communication, the fieldwork phase examines how these elements are enacted and experienced in everyday clinical practice, thereby elaborating and nuancing the theoretical components.

### Field phase

#### Attributes

The empirical material collected during the fieldwork phases [28, 29] was subsequently re-analysed using a concept analysis approach. Through this analytic process, several attributes contributing to interprofessional communication were identified (Table 2). Competence and role understanding emerged as fundamental aspects. Participants emphasised the importance of understanding both one’s own professional competence and the roles of others within the team. This ensures that each team member is aware of their responsibilities and can contribute effectively to the overall goal of patient care.

The feeling that what you do yourself when it comes to work and your profession is as important as everyone else. Like you´re a piece of the pie, and everyone else is a piece too, and together we make a whole.

Curiosity and openness were emphasised as important relational attributes. Being curious and open to different perspectives and demonstrating curiosity about others’ knowledge and experiences fostered learning, dialogue, and inclusive communication.

Good communication should partly be that… you feel more welcome if a door is open and you feel that it´s easier to go there and talk. You´re like open to the other person´s perspective as well.

Trust, cohesion, and respectful communication were described as central to interprofessional communication. Active listening, acknowledgement of competence, and respect for differing perspectives contributed to a positive communication climate.

That you feel respected…that everyone´s communication is equally important.

Collaboration and coordination were identified as components of interprofessional communication, particularly in relation to shared decision-making and managing clinical complexity.

*We consult each other often*,* just because you don´t want to sit alone with decisions.*

Moreover, clarity and adaptation in communication were emphasised. Participants highlighted the need to adapt communication to the recipient’s knowledge, role, and context to ensure shared understanding.

You must ensure that the communication is clear so that you know what to convey and that it reaches the recipient in the right way. The communication must be adapted to the person you´re communicating with.

In summary, the fieldwork phase highlights interprofessional communication as a relational and experiential process characterised by competence, curiosity, openness, trust, respect, collaboration, and communicative clarity.

Compared with the theoretical phase, the empirical findings placed stronger emphasis on relational and experiential attributes. Notably, trust, curiosity, openness, and competence emerged more prominently as actively enacted and sustained aspects of everyday interprofessional interaction, rather than primarily conceptual components.

**Table 2:** Overview of attributes from the fieldwork

Overview of attributes from the fieldwork phase	Descriptions
Competence and role understanding	Central for effective collaboration and communication among different professions, ensuring clear role understanding and contribution to patient care.
Curiosity and openness	Key to fostering a culture of learning and innovation within healthcare teams by encouraging the sharing of insights and expertise.
Trust and cohesion, along with respectful communication	Key to interprofessional communication, fostering a positive environment through active listening, perspective consideration, and skill acknowledgment. It builds trust and improves communication and patient outcomes.
Collaboration and coordination	Central to interprofessional communication, ensuring efficient information sharing and alignment toward common objectives among different healthcare professional groups.
Clarity and adaptation in communication	Central to effective interprofessional communication. Communication should be clear and tailored to the recipient´s needs, ensuring accuracy and comprehension. Facilitates smooth collaboration among healthcare professionals.

### Antecedents

Empirical data identified several antecedents shaping interprofessional communication in practice.

Leadership and clear standards were identified as essential antecedents. Leadership sets the tone for communication within the team and influences the overall communication climate. Leadership was classified as an antecedent, as it primarily shapes the conditions under which communication occurs. When leadership is clear and supportive, it fosters an environment of openness and trust among team members, facilitating effective communication and collaboration.

Organisational culture and historical context, including hierarchical traditions, influenced communication dynamics and interprofessional relationships. Awareness of these factors was described as important for navigating communication challenges.

The availability of informal communication emerged as an important antecedent. Access to informal communication channels and open-door policies facilitates communication and promotes collaboration by creating a relaxed environment for discussion. Informal communication channels provide opportunities for team members to exchange ideas and information more freely, thus improving collaboration.

In addition, transparency and information flow were identified as antecedents. Transparent, bidirectional information sharing from management to staff supports trust within the work environment and creates conditions for interprofessional communication and collaboration. When information flows openly across hierarchical and professional boundaries, it strengthens mutual trust among team members, which is vital for collaborative practice.

Lastly, hierarchy and respect were highlighted as influential antecedents. Hierarchical structures and respect for each other´s roles affect how communication works and is perceived within the team. Recognizing hierarchy while maintaining respect for each team member´s contribution fosters a positive communication climate and supports effective collaboration. To facilitate synthesis across the theoretical and empirical phases, a summary of antecedents, attributes, and consequences of interprofessional communication is presented in Table 3.

**Table 3:** Summary of antecedents, attributes, and consequences across theoretical and fieldwork phases

Analytical category	Theoretical phase	Fieldwork phase	Empirical elaboration
Antecedents	Education and knowledge, legal frameworks, logistical conditions, organisational and cultural factors, hierarchy	Leadership, transparency, informal communication, organisational culture, hierarchy	Greater emphasis on leadership practices, transparency, and informal communication
Attributes	Information sharing, collaboration, role understanding, respect, decision-making, communication tools	Competence, trust, curiosity, openness, clarity, adaptation, coordination	Stronger focus on relational and experiential attributes
Consequences	Patient safety, quality of care, team development, reduced errors	*Not explored in the fieldwork phase*	Consequences articulated mainly in theoretical literature

### The final definition – synthesis

Interprofessional communication is a purposeful, interactive process through which healthcare professionals from different disciplines exchange information, negotiate roles, and develop shared understanding to coordinate patient care. It is enacted through concrete communicative practices such as clarifying clinical responsibilities, sharing professional perspectives, engaging in joint decision-making, and adapting language to the knowledge base of other professions.

This process is characterised by key attributes, including professional competence, trust, mutual respect, role understanding, curiosity, and openness, which shape how professionals listen, respond, and collaborate in everyday clinical situations. These attributes do not exist in isolation but are continuously developed through interaction and reflected in the quality of professional relationships.

Interprofessional communication is influenced by antecedents including leadership practices, organisational structures, educational backgrounds, and hierarchical and cultural conditions. Supportive leadership, transparent communication routines, and recognition of professional expertise facilitate constructive communication, while poorly defined roles and rigid hierarchies constrain it.

Within clinical practice, particularly in psychiatric outpatient care, IPC becomes visible in both formal and informal interactions that support shared sense-making around patient needs. Effective interprofessional communication contributes to patient safety, continuity of care, and team cohesion, whereas ineffective communication increases the risk of misunderstandings, delays, and fragmented care.

This definition integrates theoretical foundations with empirical insights, defining interprofessional communication as a relational, context-dependent, and practice-oriented process central to effective healthcare delivery.

**Fig. 3:**
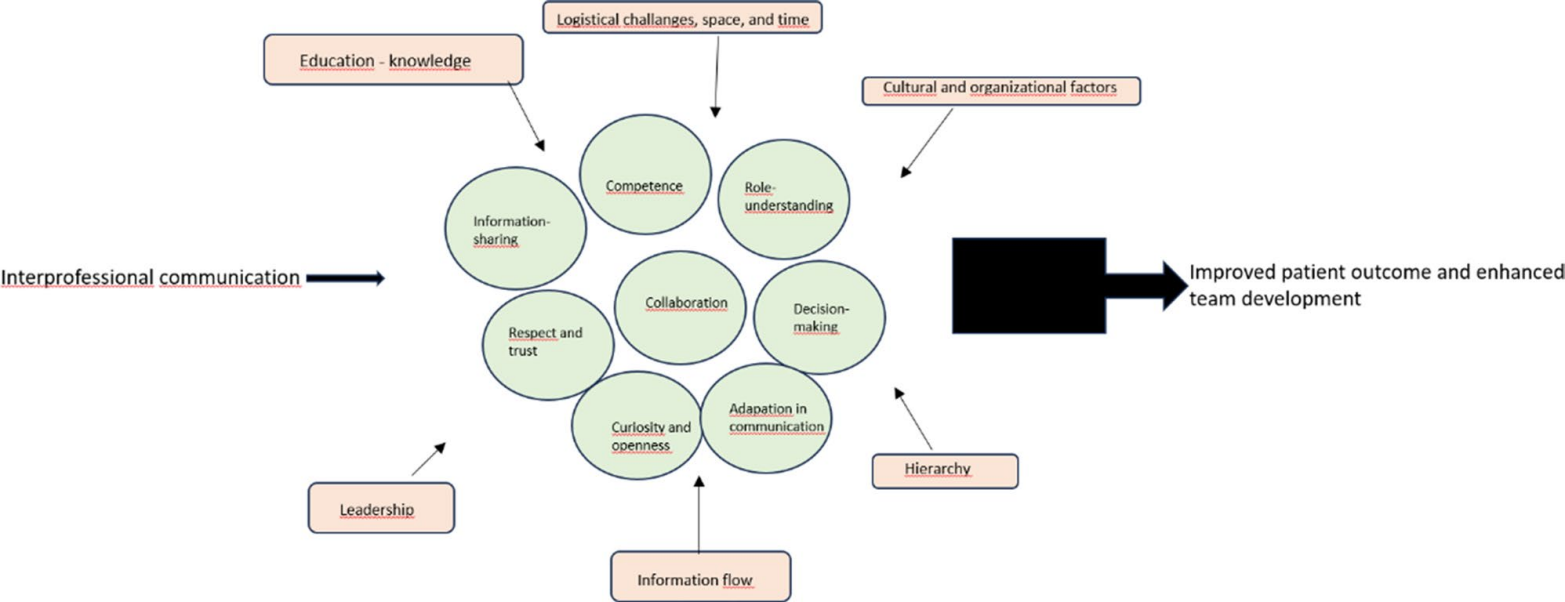
Elements of interprofessional communication. The figure illustrates how interprofessional communication develops and functions within healthcare teams. Central circles show key attributes, such as competence, respect, and trust, role understanding, curiosity, and openness, while surrounding elements represent antecedents that may influence these attributes, including leadership, cultural and organisational factors, and education. The figure shows how these attributes contribute to outcomes, indicating that effective interprofessional communication supports improved patient outcomes and enhanced team development

Figure 3, *Elements of Interprofessional Communication*, visually illustrates the interplay between attributes, antecedents, and outcomes within healthcare settings.

Schwartz-Barcott and Kim [23] suggest using Wilson´s typology to clarify conceptual boundaries. Accordingly, a model case, a contrary case, and a borderline case, drawn from clinical experiences, are shown below. The fictitious cases presented here are drawn from clinical experiences in psychiatric care and serve as an interpretation of the conceptual analysis, separate from any results found in the literature. Utilising case examples to showcase word usage variations is a comprehensive approach, emphasising the importance of clarifying concepts [74].

### Model case

In an outpatient care unit, different professionals, including physicians, nurses, assistant nurses, and rehab coordinators, work together to provide the best care possible for their patients. Recently, a patient with a history of contact with psychiatry has exhibited signs of deteriorating mental health. The patient´s contact nurse suspects that the patient has stopped taking their medication and has also recently experienced the loss of a close relative. The nurse is concerned about the patient´s well-being and wants to avoid admission to the inpatient unit. The team holds a conference to assess the situation and develop a plan to help the patient.

During the conference, the nurse uses the SBAR communication framework to provide information about the patient´s situation, background, assessment, and recommendation. Each team member shares their expertise and knowledge of the patient´s medical history, current status, and treatment plan. The use of clear and concise language, coupled with active listening and mutual respect, enhances decision-making and ultimately improves patient outcomes. The open dialogue fosters mutual respect and improves decision-making and patient outcomes. The team creates an integrated care plan and meets regularly to review the patient´s progress and adjust the treatment plan as needed. Through effective collaboration and communication, the team ensures the highest quality care for their patients.

### Contrary case

In a contrasting scenario, an assistant nurse from an outpatient psychiatric unit endeavours to address a patient´s expressed desire for talk therapy and medication reduction. However, logistical challenges hinder the assistant nurse´s attempt to discuss the matter with the team during fully booked meeting times. Seeking guidance, the assistant nurse attempts to communicate with a physician, only to encounter resistance and dismissive behaviour. The physician hastily insists on maintaining the current drug treatment, discouraging the nursing staff from getting involved, and abruptly departs without addressing the patient´s expressed interest in talk therapy. Consequently, the relevant therapeutic intervention remains unexplored. Later, it comes to light that the nurse in charge contacted the patient, fulfilling the patient´s wish for contact. Still, the comprehensive discussion on the patient´s treatment preferences and well-being was overlooked.

### Borderline case

Within an outpatient unit, a patient with a psychiatric history shows signs of deteriorating mental health. The contact nurse suspects a lack of compliance with medication, which gives rise to concerns about possible inpatient care. However, logistical challenges prevent team discussions during busy meeting times. A nurse seeks guidance, communicates with a physician, and encounters resistance and dismissive behaviour. The physician is doubtful about the current drug treatment but realises the patient´s interest in talk therapy because he lacks a clear alternative plan. At the same time, a newly involved counsellor tries to give his support but faces obstacles to being smoothly integrated into the dialogue around the patient. The charge nurse meets the patient´s need for contact, yet inadvertently overlooks a comprehensive discussion of treatment preferences, highlighting the complexities of balancing patient needs, team communication challenges, and the absence of a definitive care plan. The involvement of multiple watch professions adds another layer of interprofessional dynamics, highlighting the blurred boundaries and challenges of seamless collaboration.

## Discussion

Our study undertakes a comprehensive examination of interprofessional communication using Schwartz-Barcott and Kim´s hybrid model of concept analysis. By integrating theoretical insights with empirical data from psychiatric outpatient contexts, the model enabled a deeper exploration of the nuances shaping communication within healthcare teams. Through our examination of fundamental aspects such as competence, trust, and leadership, with a particular focus on psychiatry and various professional roles, we acknowledge the influence of contextual factors like organizational culture and hierarchical structures on communication effectiveness. Our analysis identifies opportunities to enhance interprofessional communication, emphasising the importance of role clarity and transparent information dissemination. The results enrich the conceptualization and application of interprofessional communication in the field, benefiting both healthcare clinicians and patients alike. Previous definitions of IPC emphasise information exchange and coordination between professional groups, focusing on structural or task-oriented aspects of communication. In contrast, our definition extends these perspectives by foregrounding relational and experiential dimensions such as trust, curiosity, openness, and competence as key attributes. By integrating both structural and relational elements, the present definition offers a more dynamic and context-sensitive understanding of interprofessional communication, particularly relevant to complex clinical environments (cf. [75]).

Healthcare professionals today face numerous challenges across clinical contexts, affecting their ability to work collaboratively [76]. Interprofessional communication assumes an important role in enabling collaboration by supporting open dialogue and shared understanding of goals and objectives [50, 77]. However, communication practices are shaped by contextual and organizational factors, including team composition, physical proximity, available communication tools, leadership styles, and opportunities for interaction [9, 78]. These dynamics underscore the need for organizational commitment to allocate dedicated time and space for interprofessional communication [78].

Research consistently highlights the importance of effective interprofessional communication for patient outcomes and identifies barriers such as hierarchical structures and limited opportunities for collaboration [4, 46, 48, 62]. Although effective communication is generally associated with improved performance and ineffective communication with poorer outcomes [79, 80], framing communication in binary terms risks oversimplifying its complexities and overlooking important nuances in communicative practices and their consequences.

Studies focusing on communication failures show how miscommunication can lead to misunderstandings, inadequate interventions, and medical errors, ultimately compromising patient safety [1, 48, 62, 81, 82]. Equally, insufficient collaboration and limited information flow hinder the delivery of comprehensive, patient-centred care [82]. However, simplifying communication into binary oppositions like *effective/ineffective* or *effective/ineffective” proper*/improper, oversimplifies its complexities and risks disregarding the nuances of communication. The juxtaposition of *binary oppositions* may limit our understanding of communication dynamics, failing to account for subtle variations and contextual nuances.

Consistent and transparent communication among key stakeholders in healthcare services is imperative for delivering relevant information and ensuring care consistency [83, 84]. Interprofessional communication during patient encounters plays a pivotal role in keeping healthcare professionals well-informed, thereby promoting care consistency. Conflicting advice in care plans often signifies poor interprofessional communication, triggering negative emotions among healthcare providers [85, 86]. Given the role of leadership and hierarchy as antecedents, clinical leaders are central to shaping communication climates. Leadership practices that promote transparency, psychological safety, and opportunities for informal dialogue may facilitate trust and role clarity across professional groups. At an organisational level, policies that allocate time and space for interprofessional interaction and support flattened communication structures may strengthen communication practices and collaboration.

Our analysis also illustrates the close relationship between interprofessional communication and interprofessional collaboration. While the concepts are distinct, communication forms the operational basis through which collaborative work becomes possible. Understanding this relationship is important for identifying organizational barriers and facilitators that shape cross-professional interaction. Addressing these factors at a structural level may contribute to safer work environments and align with principles of health-promoting workplaces [87].

By employing Schwartz-Barcott and Kim´s [23] hybrid model, our study advances concept development within nursing research. The combined theoretical–empirical design enabled a contextualised conceptualisation of interprofessional communication, contributing methodological depth to the field. This approach supports ongoing efforts to refine complex concepts in healthcare and strengthens the foundation for future research focused on improving communication practices across professional boundaries.

### Limitations

By integrating the phenomenological with the relativistic perspective, the study gains a deeper philosophical grounding, providing valuable tools to explore interprofessional communication in a more thoughtful and meaningful manner. However, it is important to acknowledge limitations. The relativistic view and hybrid model may not apply universally, and the focus on psychiatric outpatient clinicians could limit generalisability. While the empirical data are drawn from psychiatric outpatient settings, several identified attributes, such as trust, role clarity, and adaptive communication, are likely relevant across diverse healthcare contexts. However, how these attributes are enacted may differ in settings characterised by higher time pressure, acute decision-making, or rapid team turnover, such as emergency or acute care environments. Transferability should therefore be considered in relation to contextual similarities rather than assumed across all clinical settings. Additionally, the research methods may not capture all nuances of communication dynamics, and the study offers only a snapshot in time. Future studies and follow-ups are important for gaining further insights. Understanding these limitations is key to interpreting and applying the findings effectively.

## Conclusion

In conclusion, this study underscores the close relationship between interprofessional communication and collaboration to both patient safety and the work environment. By emphasising a cross-professional approach, interprofessional communication can be understood as a systemic process that supports safe and efficient healthcare practice. Understanding the complexities inherent in communication dynamics is important for improving communication effectiveness and promoting positive outcomes for both patients and healthcare professionals. The findings highlight the importance of cultivating interprofessional communication as a foundation for collaborative healthcare environments that support high-quality care and a sustainable work environment.

Future research could empirically test and operationalise the proposed definition of interprofessional communication in different clinical contexts. Quantitative instruments or observational frameworks could be developed to examine how specific attributes, such as shared sensemaking, role negotiation, and adaptive communication, are associated with patient outcomes, team functioning, and organisational performance. Longitudinal studies may further explore how IPC evolves over time and in response to organisational change. Continued research addressing barriers and facilitators is needed to advance understanding of its broader implications for healthcare delivery, patient safety, and healthcare professionals´ well-being.

### Relevance for clinical practice

Understanding the nuances of interprofessional communication is paramount in clinical practice. This study offers insights often overlooked, stressing role clarity and transparent information exchange. Clinicians benefit from these findings, enabling effective collaboration and enhancing patient care outcomes. Addressing communication barriers and fostering open communication cultures can bolster patient safety and cultivate supportive work environments, benefiting both clinicians and patients alike.

## Supplementary Information

Below is the link to the electronic supplementary material.

Supplementary Material 1

## Data Availability

The data generated and analysed during the present study are not publicly available due to the need to protect the participants´ integrity.
