# Supplementary material for: Interprofessional communication - a concept analysis inspired by Schwartz-Barcott and Kim´s hybrid model
Source: BMC Health Serv Res. 2026 Feb 17;26:308. doi: 10.1186/s12913-026-14180-3 (PMC12937564; doi:10.1186/s12913-026-14180-3)
Supplement: Supplementary file 1 — Supplementary Material 1 [file 12913_2026_14180_MOESM1_ESM.docx]

**Appendix 1 - Search strings used in the search**

**CINAHL**

”interprofessional communication” OR ”inter-professional communication” OR ”inter professional communication”  -649

”interprofessional communication” OR ”inter-professional communication” OR ”inter professional communication” English 627 – exported to Rayyan

**PSYCHINFO**

”interprofessional communication” OR ”inter-professional communication” OR ”inter professional communication” = 280,

”interprofessional communication” OR ”inter-professional communication” OR ”inter professional communication”, English 277 – exported to Rayyan

**WEB OF SCIENCE**

**”interprofessional communication”**(Topic) or**”inter-professional communication”**(Topic) or**”inter professional communication”**(Topic) =927

**”interprofessional communication”**(Topic) or **”inter-professional communication”**(Topic) or**”inter professional communication”**(Topic) and **English**(Languages) =889, exported to Rayyan

**SCOPUS**

( TITLE-ABS-KEY ( ”interprofessional communication” ) OR TITLE-ABS-KEY ( ”inter-professional communication” ) OR TITLE-ABS-KEY ( ”inter professional communication” ) ) 1207

( TITLE-ABS-KEY ( ”interprofessional communication” ) OR TITLE-ABS-KEY ( ”inter-professional communication” ) OR TITLE-ABS-KEY ( ”inter professional communication” ) ) AND ( LIMIT-TO ( LANGUAGE, ”English” ) ) 1147

**PUBMED**

Search: ((”interprofessional communication”[Title/Abstract]) OR (”inter-professional communication”[Title/Abstract])) OR (”inter professional communication”[Title/Abstract]) =943

Search: ((”interprofessional communication”[Title/Abstract]) OR (”inter-professional communication”[Title/Abstract])) OR (”inter professional communication”[Title/Abstract]) Filters: English 906, exported to Rayyan

**Total 3845**

**Duplicates 3456**
Using the auto-running resolve in Rayyan for 95% similarity resulted in 287 duplicates remaining - a new automated run at 94% similarity resulted in = 146 duplicates remaining, which were checked manually, resulting in **1410** articles for screening.

**Appendix 2 - Attributes Summary Table**

1. Ahmann E, Saviet M, Fouche R, Missenda M, Rosier T. ADHD Coaching and Interprofessional Communication: A Focus Group Study. INTERNATIONAL JOURNAL OF EVIDENCE BASED COACHING & MENTORING. 2021;19(2):70–87.
2. Anthoine E, Delmas C, Coutherut J, Moret L. Development and psychometric testing of a scale assessing the sharing of medical information and interprofessional communication: the CSI scale. BMC HEALTH SERVICES RESEARCH. 2014 Mar 13;14.
3. Aungst TD, Belliveau P. Leveraging mobile smart devices to improve interprofessional communications in inpatient practice setting: A literature review. Journal of Interprofessional Care. 2015 Nov 3;29(6):570–8.
4. Bardach S, Real K, Bardach D. Perspectives of healthcare practitioners: An exploration of interprofessional communication using electronic medical records. JOURNAL OF INTERPROFESSIONAL CARE. 2017 May;31(3):300–6.
5. Beyer S, Gouyet T, Letourneux V, Mener E, Huge S, Petit A, et al. Interprofessional Communication Concerning Work-Related Musculoskeletal Disorders: A Qualitative Study. JOURNAL OF OCCUPATIONAL REHABILITATION. 2018 Dec;28(4):721–9.
6. Boscart V, Heckman G, Huson K, Brohman L, Harkness K, Hirdes J, et al. Implementation of an interprofessional communication and collaboration intervention to improve care capacity for heart failure management in long-term care. JOURNAL OF INTERPROFESSIONAL CARE. 2017;31(5):583–92.
7. Brás CPDC, Ferreira MMC, Figueiredo MDCABD. Construction and validity of the Interprofessional Communication in Health Scale. Rev Bras Enferm. 2023;76(3):e20220483.
8. Bryant A, Van den Eynde M, Grewe M, Alderman J, Zomorodi M, Durham C. Interprofessional communication in the care of adults with cancer: exploring clinicians´ perceptions of team rounding. JOURNAL OF INTERPROFESSIONAL CARE. 2022 Nov 2;36(6):951–4.
9. Butler J, Fox M. Nurses´ Perspectives on Interprofessional Communication in the Prevention of Functional Decline in Hospitalized Older People. HEALTH COMMUNICATION. 2019 Jul 29;34(9):1053–9.
10. Campbell DP, Torrens C, Pollock DA, Maxwell PM. A scoping review of evidence relating to communication failures that lead to patient harm. 2018 Sep 25;2018:225
11. Carvalho K. Exploring a social pragmatic approach to overcoming barriers to interprofessional communication and across functional boundaries: A qualitative case study. Journal of Interprofessional Education & Practice. 2023 Sep;32:100649.
12. Cazeau N. Interprofessional Communication Integrating evidence to enhance systems during a pandemic. CLINICAL JOURNAL OF ONCOLOGY NURSING. 2021 Feb;25(1):56-+.
13. Choi YR, Chang SO. Exploring interprofessional communication during nursing home emergencies using the SBAR framework. Journal of Interprofessional Care. 2023 Jan 2;37(1):83–90.
14. Coifman AHM, Pedreira LC, Jesus APSD, Batista REA. Comunicação interprofissional em unidade de emergência: estudo de caso. Rev esc enferm USP. 2021;55:e03781.
15. Dean M, Gill R, Barbour J. “Let´s Sit Forward”: Investigating Interprofessional Communication, Collaboration, Professional Roles, and Physical Space at EmergiCare. HEALTH COMMUNICATION. 2016 Dec;31(12):1506–16.
16. Etheredge H, Penn C, Watermeyer J. Interprofessional communication in organ transplantation in Gauteng. Province, South Africa. SAMJ SOUTH AFRICAN MEDICAL JOURNAL. 2017 Jul;107(7):615–20.
17. Etherington C, Wu M, Cheng-Boivin O, Larrigan S, Boet S. Interprofessional communication in the operating room: a narrative review to advance research and practice. Can J Anesth/J Can Anesth. 2019 Oct 15;66(10):1251–60.
18. Fatahi N, Krupic F, Hellstrom M. Difficulties and possibilities in communication between referring clinicians and radiologists: perspective of clinicians. JOURNAL OF MULTIDISCIPLINARY HEALTHCARE. 2019;12:555–64.
19. Fernando O, Coburn NG, Nathens AB, Hallet J, Ahmed N, Conn LG. Interprofessional communication between surgery trainees and nurses in the inpatient wards: Why time and space matter. J Interprof Care. 2016 Sep;30(5):567–73.
20. Foronda C, MacWilliams B, McArthur E. Interprofessional communication in healthcare: An integrative review. Nurse education in practice. 2016 Jul;19:36–40.
21. Franz S, Muser J, Thielhorn U, Wallesch CW, Behrens J. Inter-professional communication and interaction in the neurological rehabilitation team: a literature review. Disability and Rehabilitation. 2020 May 21;42(11):1607–15.
22. Gleeson L, O´Brien GL, O´Mahony D, Byrne S. Interprofessional communication in the hospital setting : a systematic review of the qualitative literature. Journal of Interprofessional Care. 2022 Feb 2;1–11
23. Conn L, Reeves S, Dainty K, Kenaszchuk C, Zwarenstein M. Interprofessional communication with hospitalist and consultant physicians in general internal medicine: a qualitative study. BMC HEALTH SERVICES RESEARCH. 2012 Nov 30;12
24. Jafari Varjoshani N, Hosseini MA, Khankeh HR, Ahmadi F. Tumultuous Atmosphere (Physical, Mental), the Main Barrier to Emergency Department Inter-Professional Communication. GJHS. 2014 Aug 22;7(1):p144.
25. Lee L. Interprofessional communication in emergencies during a pandemic. JOURNAL OF CLINICAL NURSING. 2023 Mar;32(5–6):E3–5
26. Oosterink J, Oosterveld-Vlug M, Glaudemans J, Pasman H, Willems D, Onwuteaka-Philipsen B. Interprofessional communication between oncologic specialists and general practitioners on end-of-life issues needs improvement. FAMILY PRACTICE. 2016 Dec;33(6):727–32
27. Overbeck G, Kousgaard M, Davidsen A. Enactments and experiences of “enhanced interprofessional communication” in collaborative care-a qualitative study. JOURNAL OF INTERPROFESSIONAL CARE. 2019 Sep 3;33(5):519–27
28. Paxino J. Dynamic and distributed exchanges: an interview study of interprofessional communication in rehabilitation. Disability and Rehabilitation. 2022 Jul 8;1–11
29. Perron N, Le Breton J, Perrier-Gros-Claude O, Filliettaz S, Hudelson P, Pautex S. Written interprofessional communication in the context of home healthcare: A qualitative exploration of Swiss perceptions and practices. HOME HEALTH CARE SERVICES QUARTERLY. 2019 Jul 3;38(3):224–40
30. Rixon S, Braaf S, Williams A, Liew D, Manias E. Pharmacists´ Interprofessional Communication About Medications in Specialty Hospital Settings. HEALTH COMMUNICATION. 2015 Nov 2;30(11):1065–75
31. Smith C, Quan S, Morra D, Rossos P, Khatibi H, Lo V, et al. Understanding interprofessional communication: a content analysis of email communications between doctors and nurses. APPLIED CLINICAL INFORMATICS. 2012;3(1):38–51
32. Stewart MA. Stuck in the middle: the impact of collaborative interprofessional communication on patient expectations. Shoulder & Elbow. 2018 Jan;10(1):66–72
33. Thomas TS, Chance K, Spurlock A. Impact of Interprofessional Communication on Safety in the Neonatal Intensive Care Unit. Journal of Perinatal & Neonatal Nursing. 2023 Jul;37(3):252–60
34. Van Leijen-Zeelenberg JE, Van Raak AJA, Duimel-Peeters IGP, Kroese MEAL, Brink PRG, Vrijhoef HJM. Interprofessional communication failures in acute care chains: How can we identify the causes? Journal of Interprofessional Care. 2015 Jul 4;29(4):320–30.
35. Verhaegh K, Seller-Boersma A, Simons R, Steenbruggen J, Geerlings S, de Rooij S, et al. An exploratory study of healthcare professionals´ perceptions of interprofessional communication and collaboration. JOURNAL OF INTERPROFESSIONAL CARE. 2017 May;31(3):397–400
36. Weissenborn M, Haefeli WE, Peters-Klimm F, Seidling HM. Interprofessional communication between community pharmacists and general practitioners: a qualitative study. Int J Clin Pharm. 2017 Jun;39(3):495–506
37. Yousefi. Impact of Hospitalist Programs on Perceived Care Quality, Interprofessional Collaboration, and Communication: Lessons from Implementation of 3 Hospital Medicine Programs in Canada. JCOM [Internet]. 2021 May 28 [cited 2024 Feb 2];28(3). Available from: https://www.mdedge.com/jcomjournal/article/240518/hospital-medicine/impact-hospitalist-programs-perceived-care-quality
